# Beneficial effects of tyrosol on altered glycoprotein components in streptozotocin-induced diabetic rats

**DOI:** 10.1080/13880209.2017.1315603

**Published:** 2017-04-21

**Authors:** Ramasamy Chandramohan, Settu Saravanan, Leelavinothan Pari

**Affiliations:** Department of Biochemistry and Biotechnology, Faculty of Science, Annamalai University, Chidambaram, India

**Keywords:** Diabetes mellitus, glucose, insulin, hexose, hexosamine, sialic acid, fucose

## Abstract

**Context:** Olive oil is the major source of tyrosol which is a natural phenolic antioxidant. Olive oil constitutes a major component of the Mediterranean diet that is linked to a reduced incidence of chronic diseases.

**Objective:** This study evaluates the effects of tyrosol on altered glycoprotein components in streptozotocin-induced diabetic rats.

**Materials and methods:** Diabetes mellitus was induced in male Wistar rats by streptozotocin (40 mg/kg body weight). These rats were administered tyrosol (20 mg/kg body weight) and glibenclamide (600 μg/kg body weight) orally daily for 45 days. Plasma glucose, plasma insulin, glycoprotein components such as hexose, hexosamine, sialic acid and fucose in the plasma, liver and kidney, and histopathogy of tissues were analyzed.

**Results:** Diabetic rats revealed significant (*p* < 0.05) increase in the levels of glucose, hexose, hexosamine, sialic acid and fucose (277.17, 152.45, 100.43, 79.69 and 49.29 mg/dL) in the plasma; decrease in the levels of palsma insulin (6.12 μU/mL) and sialic acid (4.36 and 5.03 mg/g) in the liver and kidney; significant (*p* < 0.05) increase in hexose (49.33 and 46.82 mg/g), hexosamine (22.68 and 33.20 mg/g) and fucose (31.63 and 32.44 mg/g) in the liver and kidney. Further, periodic acid-Schiff staining of tissues revealed positive-stain accumulation in diabetic rats. Tyrosol treatment showed significant (*p* < 0.05) effects on all the biochemical parameters and histopathology studied in streptozotocin- nduced diabetic rats. Also, the *in vitro* study revealed the antioxidant effect of tyrosol.

**Discussion and conclusions:** Thus, tyrosol protects streptozotocin-induced diabetic rats from the altered glycoprotein components. Further, this study can be extrapolated to humans.

## Introduction

Diabetes mellitus (DM) is a leading cause of morbidity and mortality in the world’s growing population. The prevalence of DM in adults worldwide is estimated to rise from 382 million in the year 2013 to 592 million in the year 2035 (International Diabetes Federation 2013). The major part of this increase is expected to occur in developing countries, with the greatest absolute increase expected to be seen in India. According to the IDF (2013), 65.1 million people in India had DM in 2013. DM is characterized by increased blood glucose level as a result of disrupted insulin-signalling, which leads to insulin deficiency resulting from autoimmune destruction of insulin-producing β-cells in the case of type-1DM, or insulin resistance and declining β cell function in type-2 DM. The type-2 DM accounts for more than 90–95% of all cases of DM globally. It has been reported that prolonged exposure to uncontrolled chronic hyperglycemia in DM can lead to the impairment of the metabolism of glucose, lipids, proteins and glycoprotein components (Dhawan et al. 1996).

Glycoprotein is a conjugated protein covalently linked to one or more carbohydrate groups. They participate in various biological events such as cell–cell communication, protein stability, function, turnover, membrane transport, cell differentiation and recognition (Wiese et al. 1997). Thay are found on the surface of all cells and some are released into the bloodstream and body fluids, making blood and plasma more viscous (Thirunavukkarasu & Sakthisekaran 2003). It is well-documented that the carbohydrate moieties of glycoprotein components, such as hexose, hexosamine, sialic acid and fucose are hydrophilic, make glycoproteins far more hydrophilic and allows the protein to fold into proper geometry and ensure stability (Wu 2003). The carbohydrate structure of glycoprotein components is altered in many pathological conditions, including DM (Pari & Murugan 2007; Pari & Karthikesan 2009). Impaired metabolism of glycoprotein components may play a vital role in the pathogenesis of hepatic and renal diseases in DM. However, insulin deficiency during DM produces derangement of glycoprotein components metabolism, resulting in the thickening of the basal membrane of pancreatic cells. Further, an increase in the levels of glycoprotein components in diabetics indicates angiopathic complications (Konukoglu et al. 1999).

Several modern drugs are effective in preventing DM, but their prolonged use may lead to adverse effects. Hence, considerable attention has been shifted to the use of dietary constituents and natural products as an alternative or complimentary treatment for diabetic medication to reduce the adverse effects caused by the synthetic drugs. Tyrosol [4-(2-hydroxyethyl) phenol] is a well-known phenolic compound and is mainly present in extra-virgin olive oil and white wine (St-Laurent-Thibault et al. 2011). It exhibits neuro-protective, cardioprotective, anti-inflammatory, anticancer and anti-depressant effects (Bu et al. 2007; Chernyshov et al. 2007; De Stefano et al. 2007; Ahn et al. 2008; Panossian et al. 2008).

Previously reported the antihyperglycemic effect of tyrosol in streptozotocin (STZ)-induced diabetic rats and exhibits a dose-dependent response in glycaemic control (Chandramohan et al. 2015). In continuation of our research on tyrosol, in this investigation, an attempt has been made to evaluate the effects of tyrosol on plasma and tissue glycoprotein components in STZ-induced diabetic rats. The effects produced by tyrosol are compared with a standard hypoglycemic drug, glibenclamide. In addition, the *in vitro* antioxidant activity of tyrosol is evaluated to understand the mechanism of action.

## Materials and methods

### Chemicals

STZ and tyrosol were purchased from Sigma-Aldrich (St. Louis, MO). All other chemicals and solvents used were of analytical grade and purchased from Hi Media (Mumbai, India) and SD-Fine Chemicals (Mumbai, India).

### Experimental animals

Male Albino Wistar rats, weighing about 180–220 g were procured from Central Animal House, Department of Experimental Medicine, Rajah Muthiah Medical College and Hospital, Annamalai University. They were housed in clean, sterile, polypropylene cages under standard vivarium conditions (12 h light/dark cycles) with free access to standard chow (Hindustan Lever Ltd., Bangalore, India) and water. The experimental protocol was approved by the Institutional Animal Ethical Committee, Annamalai University (Reg No. 1002, 2013).

### Induction of experimental DM

DM was induced in overnight fasted rats by a single intraperitoneal injection of STZ (40 mg/kg body weight) dissolved in freshly prepared citrate buffer (0.1 M, pH 4.5). STZ-injected rats were allowed to drink 20% glucose solution overnight to overcome the initial drug-induced hypoglycemic mortality. The induction of DM in rats was confirmed by estimating the elevated plasma glucose levels, 72 h after STZ injection by the method of Trinder (1969). Rats with fasting plasma glucose levels more than 250 mg/dL were considered diabetic and chosen for the current study (Chandramohan et al. 2015).

### Experimental design

A total of 30 rats (18 STZ-induced diabetic rats and 12 normal rats) were used and they were divided into five groups with six rats in each group as follows:

Group I: normal control rats.

Group II: normal rats given intragastrically 1 mL of tyrosol (20 mg/kg body weight)dissolved in distilled water daily for 45 days.

Group III: STZ-induced diabetic control rats.

Group IV: STZ-induced diabetic rats treated with 1 mL of tyrosol (20 mg/kg body weight) intragastrically dissolved in distilled water daily for 45 days (Chandramohan et al. 2015).

Group V: STZ-induced diabetic rats treated with 1 mL of glibenclamide (600 μg/kg body weight) intragastrically dissolved in distilled water daily for 45 days (Chandramohan et al. 2015).

At the end of the experimental period, the rats were deprived of food overnight, anesthetized intramuscularly using ketamine (24 mg/kg body weight) and sacrificed by cervical decapitation. The blood samples were collected from the rats for the estimation of plasma glucose, insulin and glycoprotein components. Liver and kidney tissues were dissected out immediately, washed in ice-cold saline, patted dry and weighed for histology.

### Biochemical estimations

#### Extraction of glycoprotein components

To 0.1 mL of plasma, 5.0 mL of methanol was added, mixed well and centrifuged for 10 min at 3000*g*. The supernatant was decanted and the precipitate was again washed with 5.0 mL of 95% ethanol, recentrifuged and the supernatant was decanted to obtain the precipitate of glycoprotein components. This was used for the estimation of hexose, hexosamine, fucose and sialic acid in plasma.

For the extraction of glycoprotein components from the tissues (liver or kidney), a known weight of the tissue was homogenized in 7.0 mL of methanol. The contents were filtered and homogenized with 14 mL of chloroform. This was filtered and the residue was successively homogenized in chloroform:methanol (2:1v/v) and each time the extract was filtered. The residue (defatted tissues) was obtained and the filtrate was decanted. A weighed amount of defatted tissue was suspended in 3.0 mL of 2 N HCl and heated at 90 °C for 4 h. The sample was cooled and neutralized with 3.0 mL of 2 N NaOH. Samples from this were used for the estimation of hexose, hexosamine, sialic acid and fucose in tissues (Stanely Mainzen Prince & Kannan 2006; Sundaram et al. 2012).

#### Determination of plasma glucose and insulin levels

Plasma glucose levels were estimated by a commercial kit (Sigma Diagnostics Pvt. Ltd., Baroda, India) by the method of Trinder (1969). Plasma insulin was assayed using ELISA kit (Boeheringer–Manneheim Kit, Manneheim, Germany).

#### Determination of glycoprotein components levels

Hexose was estimated by the method of Niebes (1972). The reaction mixture contained 0.5 mL of tissue homogenate/plasma, 0.5 mL of 5% phenol and 2.5 mL of conc. H_2_SO_4_ and boiled for 20 min, and absorbance was read at 490 nm.

Hexosamine was estimated by the method of Elson and Morgan (1933) with slight modifications by Niebes (1972). Briefly, the reaction mixture contained 0.5 mL plasma/1.0 mL tissue homogenate and 2.5 mL of 3 N HCl. It was boiled for 6 h and neutralized with 6 N NaOH. To 0.8 mL of the neutralized sample, 0.6 mL of acetyl acetone reagent was added and boiled for 30 min. The mixture was treated with 2.0 mL of Ehrlich’s reagent. The colour developed was read at 540 nm colorimetrically.

Sialic acid was determined by the method of Warren (1959). In brief, 0.5 mL of tissue homogenate/plasma was treated with 0.5 mL of de-ionized water and 0.25 mL of periodic acid and incubated at 37 °C for 30 min. 0.2 mL of sodium meta-arsenate and 2.0 mL of thiobarbituric acid were added to the reaction mixture which was heated for 6 min. 5.0 mL of acidified butanol was then added and the absorbance was read at 540 nm.

Fucose was estimated by the method of Dische and Shettles (1948) 0.5 mL of tissue homogenate/plasma was treated with 4.5 mL of H_2_SO_4_ and boiled for 3 min. Cysteine hydrochloride reagent (0.1 mL) was then added. After 75 min in the dark, the absorbance was read at 393 and 430 nm. The glycoprotein levels were expressed as mg/100 g for defatted tissue and mg/dL for plasma.

### Histopathology

#### Periodic acid-schiff (PAS) stain

Normally PAS stain is used to identify glycoprotein components by histopathological examination (Kumar & Salimath 2014). The liver and kidney tissues were kept in 10% formalin immediately after their removal and sections of 5 μm thickness were taken after series of alcohol (70–100%) washings and paraffinization. After deparaffinization and hydration, sections were placed in 1% periodic acid for 15 min followed by water wash and Schiff’s reagent treatment, followed by staining with Mayer hematoxylin.

### The in vitro study

#### Total antioxidant activity

The total antioxidant potential of tyrosol was determined by the 2, 2′-azinobis-(3-ethyl-benzothiazoline-6-sulfonic acid) radical (ABTS^•^+) assay, as described by Miller et al. (1996). The reaction mixture contained ABTS (0.002 M), tyrosol (25–150 μM) and buffer in a total volume of 3.5 mL. The absorbance was measured at 734 nm in a Systronics UV-visible Spectrophotometer.

### Statistical analysis

Data are presented as means ± standard deviation (SD) and subjected to statistical significance, then were evaluated by one-way analysis of variance (ANOVA) using Statistical Package for the Social Sciences (SPSS) software package version 16.0 (SPSS, Cary, NC) and the individual comparisons were obtained by Duncan’s Multiple Range Test (DMRT). Values are considered statistically significant when *p <* 0.05.

## Results

### Effect of tyrosol on plasma glucose and plasma insulin levels

**Figure 1.**
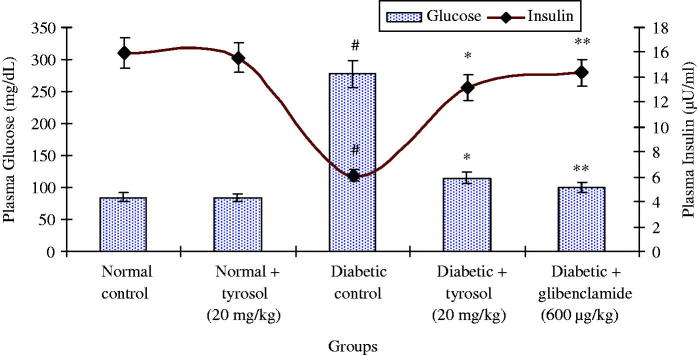
Changes in the levels of plasma glucose and insulin. Each column is mean ± SD for six rats in each group. Values are statistically significant at *p <* 0.05 (DMRT), when compared with (#) normal control and normal + tyrosol treated groups (* and **) diabetic control groups.

Figure 1 reveals the levels of plasma glucose and insulin in normal control and experimental rats. The level of fasting plasma glucose was significantly (*p* < 0.05) increased and the level of plasma insulin was significantly (*p* < 0.05) decreased in STZ-induced diabetic control rats as compared to normal control rats. Treatment with tyrosol (20 mg/kg body weight) daily for a period of 45 days to diabetic rats showed a significant (*p* < 0.05) decrease in the level of plasma glucose and a significant (*p* < 0.05) increase in the level of plasma insulin when compared to STZ-induced diabetic control rats (Figure. 1). Normal control and tyrosol-treated group did not show any significant changes in plasma glucose and insulin levels.

### Effect of tyrosol on plasma glycoprotein components

**Figure 2.**
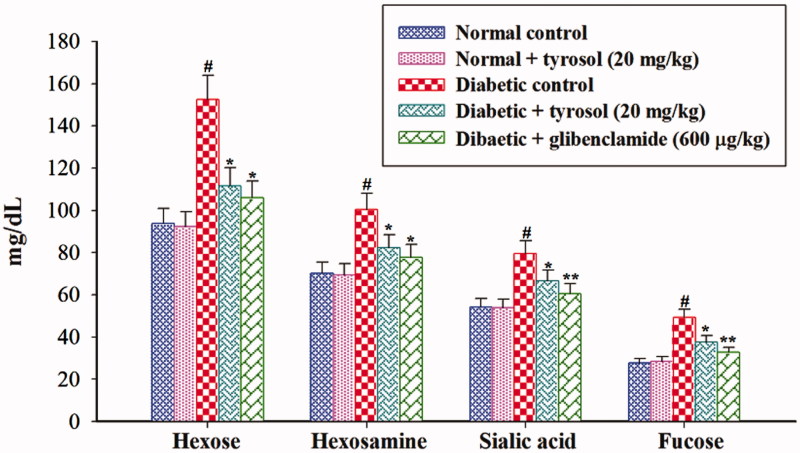
Changes in the levels of plasma glycoprotein components. Each column is mean ± SD for six rats in each group. Values are statistically significant at *p <* 0.05 (DMRT), when compared with (#) normal control and normal + tyrosol treated groups (* and **) diabetic control groups.

Figure 2 shows the changes in the levels of plasma hexose, hexosamine, sialic acid, and fucose in normal control and experimental rats. There was a significant (*p* < 0.05) increase in the levels of plasma hexose, hexosamine, sialic acid, and fucose in STZ-induced diabetic control rats compared to normal control rats. Tyrosol and glibenclamide treatment to STZ-induced diabetic rats revealed a significant (*p* < 0.05) reduction of hexose, hexosamine, sialic acid and fucose in the plasma when compared to STZ-induced diabetic control rats. Normal control and tyrosol-treated group did not show any significant changes in plasma glycoprotein components.

### Effect of tyrosol on tissue glycoprotein components

**Figure 3.**
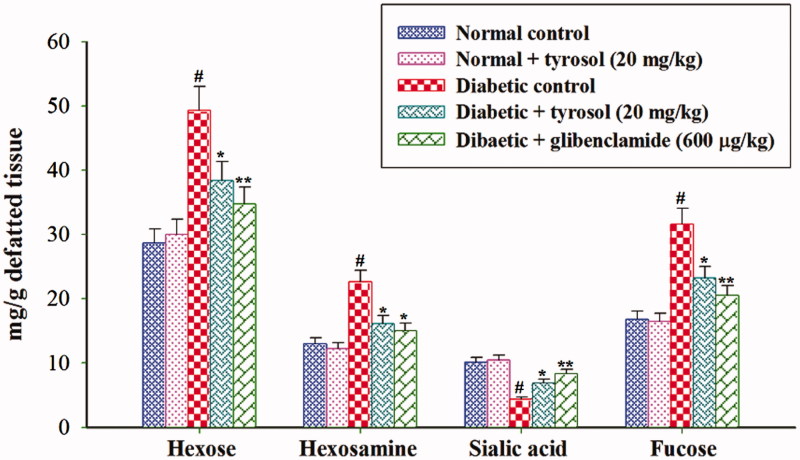
Changes in the concentration of liver glycoprotein components. Each column is mean ± SD for six rats in each group. Values are statistically significant at *p <* 0.05 (DMRT), when compared with (#) normal control and normal + tyrosol treated groups (* and **) diabetic control groups.

**Figure 4.**
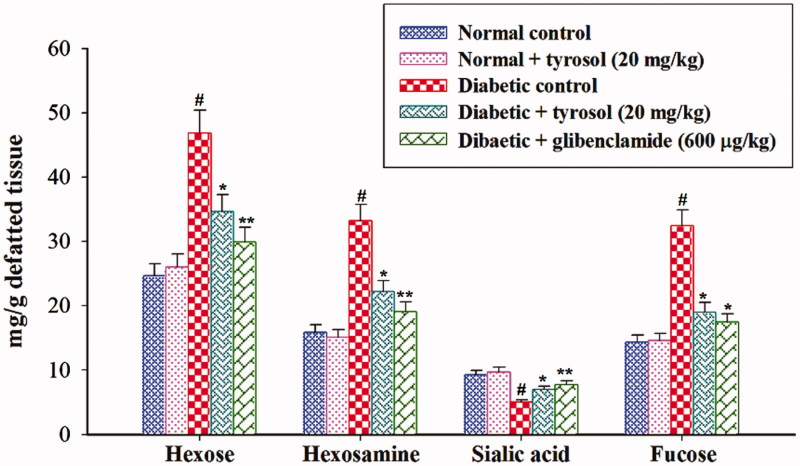
Changes in the concentration of kidney glycoprotein components. Each column is mean ± SD for six rats in each group. Values are statistically significant at *p <* 0.05 (DMRT), when compared with (#) normal control and normal + tyrosol treated groups (* and **) diabetic control groups.

The concentrations of hexose, hexosamine and fucose were significantly (*p* < 0.05) increased in the liver and kidney whereas the concentration of sialic acid was significantly (*p* < 0.05) decreased in the liver and kidney of STZ-induced diabetic control rats compared to normal control rats. Tyrosol and glibenclamide treatment significantly (*p* < 0.05) decreased the concentration of hexose, hexosamine, fucose and significantly (*p* < 0.05) increased the concentration of sialic acid in the liver and kidney of STZ-induced diabetic rats when compared to STZ-induced diabetic control rats (Figures 3 and 4). Normal control and tyrosol-treated group did not show any significant changes in tissue glycoprotein components.

### Histopathological study

**Figure 5.**
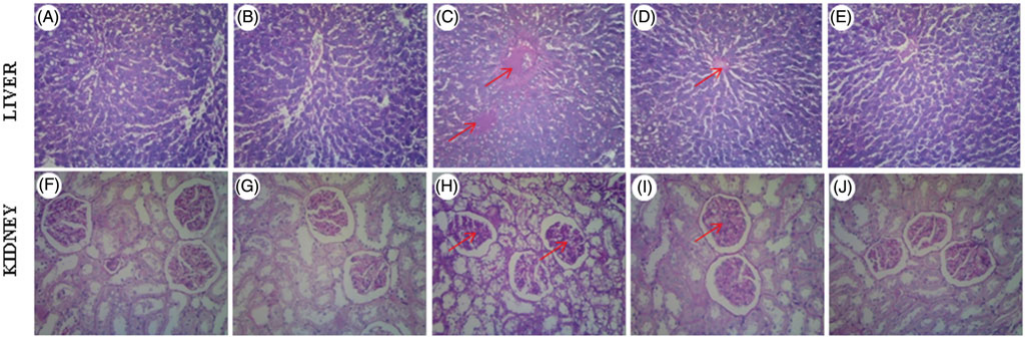
(A–J) Histopathology of rat liver and kidney sections stained with PAS. (A,F) normal control rats (100 X), (B,G) normal rats treated with tyrosol (100 X), (C,H) diabetic control rats(100 X), (D,I) diabetic rats treated with tyrosol (100 X), (E,J) diabetic rats treated with glibenclamide (100 X).

Histopathological observations of the experimental rat’s liver and kidney sections stained with PAS are shown in Figure 5(A–J). Normal control rats showed normal pattern for PAS staining (A, F), tyrosol-administered (20 mg/kg body weight) normal rats also showed normal pattern for PAS staining (B, G), diabetic control rats revealed increase PAS-positive stain around the central vein (C, H), tyrosol-treated (20 mg/kg body weight) diabetic rats revealed significantly reduced PAS-positive stain (D, I), glibenclamide-treated (600 μg/kg body weight) diabetic rats revealed markedly reduced accumulation for PAS-positive stain (E, J).

### Effect of tyrosol on total antioxidant activity

**Figure 6.**
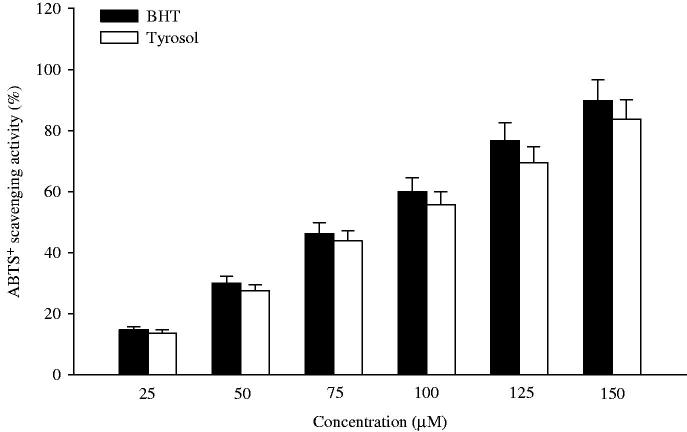
The *in vitro* total antioxidant activity of tyrosol. Columns are the average of triplicate experiments.

The *in vitro* total antioxidant activity of tyrosol was evaluated by ABTS^•^+ scavenging method. Inhibition of ABTS^•^+ showed concentration-dependent (25, 50, 75, 100, 125 and 150 μM) scavenging activity of tyrosol (Figure 6). The percentage scavenging activity of tyrosol increases with increasing concentration. However, the highest percentage (83.73%) scavenging activity of tyrosol was observed at 150 μM concentration. The effect of tyrosol *in vitro* was compared with a standard, butylated hydroxytoulene (BHT).

## Discussion

STZ has been extensively used to induce DM in experimental rat models. The intraperitoneal administration of STZ (40 mg/kg body weight) causes partial destruction of pancreatic β-cells in rats that decrease the synthesis and secretion of insulin and causes hyperglycemia (Punithavathi et al. 2011; Sheikh et al. 2015). The increased levels of plasma glucose and decreased levels of insulin observed in STZ-induced diabetic rats were brought back to near normal by tyrosol treatment. We previously reported that tyrosol treatment significantly decreased plasma glucose, glycosylated hemoglobin and significantly increased plasma insulin, total hemoglobin and body weight in STZ-induced diabetic rats. The activities of key carbohydrate metabolic enzymes such as phosphoenolpyruvate carboxykinase, fructose-1, 6-bisphosphatase and glucose-6-phosphatase were significantly decreased and the activities of hexokinase and glucose-6-phosphate dehydrogenase were significantly increased in the liver and kidney of STZ-induced diabetic rats treated with tyrosol. Further, a significant increase in glycogen content in the liver and muscle, glycogen synthase activity in the liver and a significant decrease in the activity of liver glycogen phosphorylase were observed in diabetic rats treated with tyrosol. Tyrosol may have enhanced the insulin secretion from remnant pancreatic β cells, which in turn enhanced glucose utilization by peripheral tissues of diabetic rats either by promoting glucose uptake and metabolism, or by inhibiting hepatic gluconeogenesis, thereby decreasing blood glucose levels (Chandramohan et al. 2015).

In diabetic state, abnormalities of glycoprotein metabolism are commonly observed (Anil Kumar et al. 2005). We observed elevated levels of hexose, hexosamine, sialic acid and fucose in the plasma of STZ-induced diabetic control rats. The secretion or shedding from cell membrane glycoconjugates into the circulation leads to the elevation of plasma glycoprotein components. Also, insulin deficiency and high levels of plasma glucose in diabetic condition may result in an increased synthesis of glycoprotein components (Patti et al. 1999). Tyrosol and glibenclamide treatment to STZ-induced diabetic rats significantly decreased plasma glycoprotein components to near normal levels, by virtue of its antihyperglycaemic effects.

Sialic acid can be used as a measurement of the acute phase response because many of these glycoproteins have sialic acid as the terminal, non-reducing positions of carbohydrate chains on the outer and inner membrane surfaces (Pickup 2004). Sialic acid levels on glycoproteins are regulated by sialyltransferases during their cellular biosynthesis and, in some instances, by sialidase(s) after secretion from cells. A decrease in sialyltransferase and an increase in sialidase activities (Chari & Nath 1984; Cohen-Forterre et al. 1990) as well as changes in other glycosidase activities (Serrano et al. 1983) have been reported in humans and animals suffering from DM. Also, diabetic rats exhibited increased levels of sialic acid in the plasma and decreased concentration in tissues (Pari & Murugan 2007). The decreased level of sialic acid is observed in the diabetic rat’s tissues might be due to increased synthesis of fibronectin, which contains sialic acid in its core structure (Basha & Sankaranarayanan 2015). Further, the decreased tissue sialic acid level is associated with oxidative stress-induced desialylation of glycoproteins (Goswami & Koner 2002). In our study, tyrosol treatment normalized sialic acid levels in the plasma and tissues of diabetic rats, which could be due to the regulation of sialidase activity, by virtue of its antihyperglycaemic effects.

Hexosamine a nitrogenous sugar in which an amino group replaces a hydroxyl group. The level of hexosamine increased significantly in the plasma and tissues of diabetic rats, which may be due to insulin deficiency. Further, the concomitant oxidative stress increases the expression of GFAT (Glutamine: Fructose 6-phosphate amino transferase), the rate-limiting enzyme of this pathway leading to higher hexosamine levels (Brownlee 2005). Protein-bound hexose in the cell membrane provides hydrophobic nature. In this study, we observed increased levels of hexose in the plasma and tissues of diabetic rats, which may be due to depressed utilization of glucose by insulin-dependent pathway, thereby enhancing the formation of hexose and hexosamine for the accumulation of glycoproteins (Patti et al. 1999). Treatment with tyrosol and glibenclamide significantly lowered hexose and hexosamine levels, which might be due to its antihyperglycaemic effects.

Fucose is a member of a group of eight essential sugars that the body requires for the optimal functioning of cell-to-cell communication and its metabolism appear to be altered in various diseases such as DM (Mondoa & Kitei 2001). Wiese et al. (1997) suggested that serum and hepatic fucosyltransferase and fucosidase activities are increased in STZ-induced diabetic rats. Fucosylation reactions confer unique functional properties to glycoproteins. The observed increased concentration of liver and kidney fucosylated proteins in STZ-induced diabetic control rats might be due to increased synthesis and decreased degradation of this protein. Tyrosol treatment to STZ-induced diabetic rats significantly decreased fucose concentration, which may be due to the regulation of the fucosylated protein levels, by its antihyperglycaemic effects.

Also, confirming the biochemical findings, we carried out PAS staining of liver and kidney. The PAS stained sections revealed glycoproteins accumulation in the liver and kidney of diabetic rats. Tyrosol treated diabetic rats showed near normal liver and kidney morphology, which indicate that tyrosol decreased glycoprotein components accumulation in STZ-induced diabetic rats.

Furthermore, we studied *in vitro* scavenging effect of tyrosol on ABTS^•^+. The decolorization of ABTS^•^+ cation radical is an unambiguous way to measure the antioxidant activity of phenolic compounds. Our study revealed that tyrosol-scavenged ABTS^•^+ concentration dependently. Tyrosol at the concentration of 150 μM showed the highest scavenging effect compared to the other five doses (25, 50, 75, 100 and 125 μM). Our results revealed definite scavenging activity of the tyrosol towards ABTS^•^+ in comparison with butylated hydroxytoulene (BHT). Thus, tyrosol is a potent antioxidant.

In conclusion, tyrosol treatment reduced accumulation of glycoprotein components in STZ-induced diabetic rats in addition to its antidiabetic effect. Tyrosol also exhibited *in vitro* antioxidant property. The observed effect of tyrosol on decreasing the adverse effects of hyperglycemia provides an insight into the pathogenesis of diabetic complications, and may be used in therapeutic approaches.
